# Evaluation of the Role of a Hybrid Filler of Hyaluronic Acid and Calcium Hydroxyapatite in the Management of Post‐Acne Scars Using 2D and OCT Digital Morphometry

**DOI:** 10.1111/jocd.16768

**Published:** 2025-01-16

**Authors:** Ilaria Proietti, Emanuele Amore, Camilla Chello, Federica Trovato, Francesca Paola Sasso, Giovanni Pellacani, Stefania Guida, Concetta Potenza

**Affiliations:** ^1^ Dermatology Unit Daniele Innocenzi A. Fiorini Hospital Terracina Italy; ^2^ Dermatologic Unit, Department of Clinical Internal, Anesthesiological and Cardiovascular Sciences La Sapienza University of Rome Rome Italy; ^3^ School of Medicine, Clinic of Dermatology, IRCCS San Raffaele Hospital Vita‐Salute San Raffaele University Milan Italy

## 1 Introduction

Acne scars are a frequent consequence of severe acne, characterized by depressions (atrophic) or raised areas on the skin (hypertrophic or keloid). These scars represent the abnormal outcome of wound healing, resulting in altered collagen production. Treatment options depend on the type and severity of the scars and may involve topical treatments, chemical peels, laser therapy, dermal fillers, and the use of botulinum toxin [1]. The choice of a proper treatment may reduce and even resolve the cutaneous disorder, leading to a refinement in skin texture and appearance, improving patients' confidence and dermatology quality life index (DLQI). Early treatments are essential to reduce long‐term scarring [2, 3].

## 2 Case Presentation

We present the case of a 30‐year‐old woman with Fitzpatrick skin phototype II, who came to our Department to manage post‐acne scars. She was treated with oral isotretinoin with partial response and already tried peeling, such as salicylic and glycolic ones, and pulsed light, without benefits. At clinical observation, the patient presented depressions and erythematous lesions on the zygomatic areas bilaterally.

We decided to use a hybrid injectable, combining hyaluronic acid (HA) and calcium hydroxyapatite (CaHA), in a 1.25 mL prefilled syringe with lidocaine hydrochloride (3 mg/mL) with 27G 1/2″ thin‐walled sterile injected in subcutaneous layer by 23G 40 mm cannula. A cannula, with its flexible and blunt tip, significantly reduces the chances of puncturing blood vessels, thereby lowering the risk of serious complications such as tissue necrosis or visual disturbances. Additionally, using a cannula can minimize pain, swelling, and bruising, leading to a safer and more satisfactory aesthetic result. We performed the procedure at T0 and a follow‐up visit after 12 weeks (T1). At T0 and T1, we collected two‐dimensional (2D) photos (VISIA) and optical coherence tomography (OCT) images.

**FIGURE 1:**
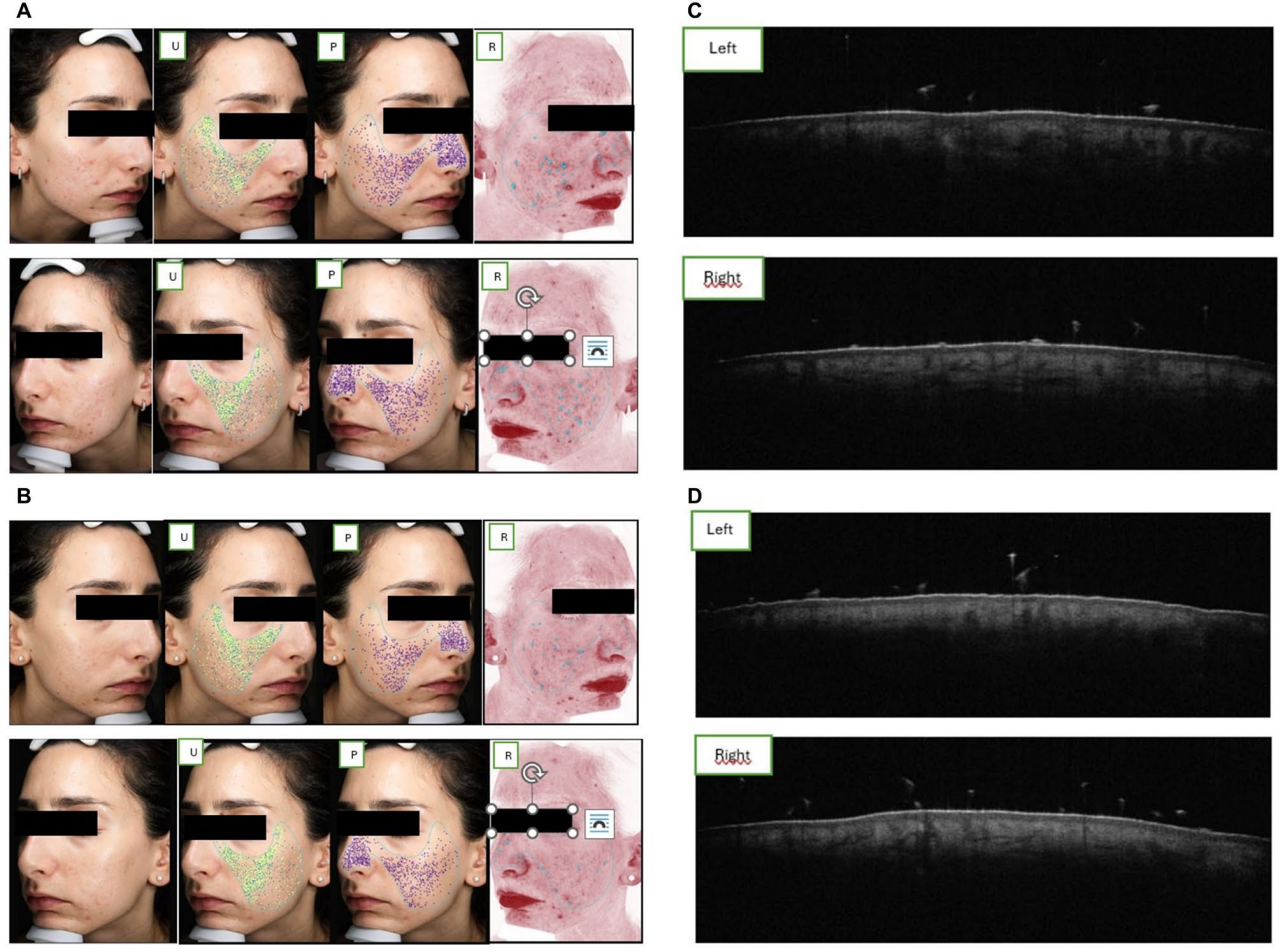
(A) 2D photographs (T0). (B) 2D photographs (Tw12). (C) OCT at T0. (D) OCT at week 12 (T1). (C, D) Increased dermal density and collagen deposition from baseline (T0) to week 12 (T1). Collagen fibers are more compact with a uniform and sustained structure.

In 2D photos, we found a smoother appearance of the skin, with reduced scar depth and improved texture from baseline. The VISIA analysis showed that pores (P), redness (R), and uniformity (U) of the skin on the left and right cheeks improved from T0 to T1 (Table 1; Figure 1B).

**TABLE 1:** VISIA measurements.

	Left cheek T0 (absolute value)	Left cheek T1 (absolute value)	Right cheek (absolute value)	Right cheek (absolute value)
Pores (T0)	726	622	748	557
Uniformity (T0)	1334	1488	1270	1472
Redness (T0)	36	13	31	17

OCT analysis conducted on the left and right cheeks at T0 and T1 revealed an improvement in scar depth and increased dermal thickness. In addition, increased rarefaction of the dermis can be seen at T0, which regains firmness after hydroxyapatite administration. Collagen density increased after 12 weeks (Table 2; Figure 1C,D).

**TABLE 1:** ROI1 statistics.

	Left cheek	Right cheek
Density (T0)	45.818582	51.805556
Density (T1)	71.130780	66.705487
Attenuation (T0)	0.001749	0.001613
Attenuation (T1)	0.002899	0.002234

Additionally, a reduction of DLQI from 25 to 5 was registered, therefore revealing an improvement in the quality of life.

## 3 Discussion

In the field of acne scar treatment, the decision‐making process for choosing the appropriate injectable must be tailored to the specific needs of the patient's skin. For our patient, the goal was to reorganize the fibrillar collagen fibers in the papillary dermis into a linear and ordered pattern, to reconstruct the skin's structure, and to regenerate the dermoepidermal junction (DEJ). We selected an injectable based on a combination of hyaluronic acid (HA) and calcium hydroxyapatite (CaHA) to promote fibroblast differentiation, stimulate new collagen formation, and restore skin plumpness [4, 5, 6]. The cross‐linked HA‐based gel can produce an immediate lifting effect. As the carrier gel degrades over time, a solid and dense collagen structure forms in its place, providing a gradual, long‐lasting lifting effect associated with CaHA. Moreover, data from literature highlight that patients treated with the integrated HA approach showed significant improvement in skin hydration, reduction in redness, and overall better skin condition and improvement of DLQI [7, 8].

## 4 Conclusion

We strongly believe that selecting the right filler for each individual patient is crucial for achieving successful treatment outcomes. In conclusion, the hybrid filler composed of hyaluronic acid and calcium hydroxyapatite seems to be very promising in managing post‐acne scars. It effectively enhances clinical appearance and promotes tissue remodeling while maintaining a high safety profile with no adverse effects.

## 5 Author Contributions

All authors equally contributes to the article.

## 6 Conflicts of Interest

The authors declare no conflicts of interest.

7

## Data Availability

The data that support the findings of this study are available on request from the corresponding author. The data are not publicly available due to privacy or ethical restrictions.
